# Effects of aerobics training on anxiety, depression and sleep quality in perimenopausal women

**DOI:** 10.3389/fpsyt.2022.1025682

**Published:** 2022-11-24

**Authors:** Yan Zhao, Hualiang Niu, Shengjie Liu

**Affiliations:** ^1^Department of Physical Education, Lyuliang University, Lvliang, China; ^2^Department of Physical Education, Changzhi University, Changzhi, China

**Keywords:** aerobics training, perimenopausal women, anxiety, depression, sleep quality

## Abstract

**Objective:**

To investigate the effect of aerobics training on anxiety, depression and sleep disturbance in perimenopausal women.

**Materials and methods:**

A total of 289 perimenopausal women with anxiety, depression or sleep disorder in Lishi district of Luliang city were treated with aerobics training. Self-rating anxiety scale, self-rating depression scale and Pittsburgh sleep scale were used to investigate the anxiety, depression and sleep status of perimenopausal women before and after intervention.

**Results:**

After aerobics training, the scores of self-rating anxiety scale, self-rating depression scale and Pittsburgh sleep questionnaire were 48.26 ± 6.47, 50.27 ± 6.54 and 10.64 ± 4.38, respectively. The levels of anxiety, depression and sleep disorder in 289 subjects were significantly lower than those before intervention (*t* = 3.865, 4.541, 5.596, *P* < *0.01*). The remission rate of symptoms was significantly different in subjects with different frequency of spontaneous practice (*P* < *0.01*), and the higher the number of spontaneous practice, the higher the remission rate of anxiety, depression and sleep disorders in subjects (*P* < *0.01*).

**Conclusion:**

aerobics training can effectively improve the anxiety and depression of perimenopausal women, improve sleep quality, the more frequency of practice, the more obvious the effect. It can be used as an intervention to improve the mental health level of perimenopausal women in terms of anxiety, depression and sleep quality in clinical promotion.

## Introduction

At present, Chinese society has entered the era of aging population, with the aging process of the population aggravated and accelerated, more and more women into the perimenopausal period. Perimenopausal period refers to the period from the onset of endocrine, biological, and clinical features associated with menopause near menopause to within 1 year of menopause (1). During this period, due to the decline of ovarian function, the level of estrogen progesterone decreases in a cliff-like manner, the number and quality of oocytes decrease, and the levels of estrogen and progesterone continuously decline (2). Some women in this period may not only be prone to menstrual disorders, hot flashes, sweating, insomnia, palpitations, vertigo, fatigue, headache, frequent urination, and muscle aches throughout the body, pain and discomfort in the bone and joint parts of the body, emotional instability and other physiological symptoms, but also easily associated with anxiety, depression and other mental disorders (3). Foreign studies have shown that after women enter perimenopausal period, the risk of anxiety and depression is significantly higher (4), psychological distress and mood disorders significantly affect the physical health and quality of life of perimenopausal women (5). Previous treatments for mood disorders in perimenopausal women have mostly been menopausal hormone therapy and drug therapy (6), but hormone and drug therapy have many contraindications, and long-term use of drugs can also produce many adverse reactions (7). In addition to hormone and drug therapy, there are also studies on intervention through psychological care, and the method of intervention is mainly health education or through talking to patients, using external interventions such as language, attitude, expression, and behavior to affect the patient’s understanding and mood. However, these methods also have certain defects, for example, there are no specific operating specifications, reproducibility is not strong, and the therapeutic effect cannot be ensured. Physical exercise has been recognized as a psychotherapeutic approach as a low-cost, complication-free, non-invasive non-pharmacological therapy (8). According to a survey in the United States, 80% of 1,750 psychologists believe that physical exercise is one of the effective treatments for depression, and 60% believe that physical exercise should be used as a treatment to eliminate anxiety (9). Among college students, many suffer from anxiety and depression due to learning and other setbacks, which can be slowed or eliminated by physical exercise (10). It has also been confirmed that moderate exercise has a significant anti-insomnia effect and has a positive regulatory effect on the sleep-wake cycle, which can replace sedative hypnotic drugs to a certain extent without side effects. Moreover, exercise training improves quality of life and mood while relieving sleep disturbances (11). It has been suggested that physical exercise may help to avoid them before mental health problems begin (12). It has also been shown that exercise improves symptoms in many existing psychiatric disorders (13).

Aerobics is an emerging sports program that integrates gymnastics, dance, music, is based on aerobic exercises, and is characterized by health, strength, and beauty (14, 15). Existing research suggests that women’s participation in mass aerobics can not only maintain positive and optimistic mood and improve mood, but also interfere with depression and anxiety. Beautiful and cheerful music rhythm and lively and pleasant physical movements can maximize people’s enthusiasm and vitality, so that the tired mind can be effectively relaxed and the body and mind can be fully regulated (16, 17). However, there is no research report on the effect of aerobics training on anxiety, depression and sleep quality in perimenopausal women. In this study, 8-week aerobics training was used to intervene perimenopausal women with anxiety, depression and insomnia to explore the effect of aerobics training in the treatment of mood disorders in perimenopausal women. It is expected to establish a psychological intervention method that can effectively alleviate the adverse emotions of perimenopausal women, is easy to operate, and is convenient for promotion.

## Materials and methods

### Study subjects

In April 2022, using simple random sampling, total of 2,076 women aged 45–55 years old in Chengdong Community, Fengshan Ecological Park Community and Bayi Community of Lianhuachi Street in Binhe Street, Lishi District, Luliang City were investigated by perimenopausal symptoms questionnaire. After the respondents signed the informed consent, the investigators used structured questionnaires to investigate the respondents. The questionnaire was designed with the question form method. To ensure the quality of the questionnaire, the questionnaire was filled out with detailed instructions and explanations from the investigators before filling in the questionnaire. After the informed consent of the respondents, the concerns of privacy disclosure and other aspects were eliminated, and the active cooperation of the respondents was obtained. For those with low education level who could not complete the questionnaire independently, the investigators read each item and the respondents checked the corresponding options after thinking independently. After the completion of the return are reviewed, the missing items face to face explanation and ask to fill back. A total of 974 women with anxiety, 839 women with depression and 1,505 women with insomnia (including the coexistence of three symptoms) were screened. According to the inclusion and exclusion criteria, 289 participants were finally identified, including 39 participants with anxiety only, 43 participants with depression only, 51 participants with insomnia only, 38 participants with anxiety and depression, 42 participants with anxiety and insomnia, 52 participants with depression and insomnia, and 24 participants with anxiety and depression and insomnia. The total number of patients with anxiety was 143, with an average age of 51.4 ± 4.7 years; the total number of patients with depression was 157, with an average age of 51.7 ± 4.3 years; and the total number of patients with insomnia was 169, with an average age of 52.2 ± 4.6 years. There was no significant difference between the ages of each group (*F* = 1.25, *P* = 0.29). Inclusion criteria: (1) Perimenopausal symptoms (menstrual disorders, vertigo, palpitations, headache, tinnitus, facial flushing, anxiety, depression, insomnia, etc.) in the past 6 months, in line with perimenopausal diagnosis. (2) Junior high school degree or above, have certain ability to read and understand words, be able to follow the command and be able to fill in the questionnaire independently. (3) Healthy and no history of skeletal-related diseases. (4) all study subjects had no relevant special training basis at the beginning of this training. (5) Voluntary participation in this study. Exclusion criteria: (1) Suffering from mental illness and severe vision, hearing impairment. (2) Patients with heart, lung, liver, kidney and other organ diseases and endocrine system diseases, malignant tumors. (3) Insomnia, anxiety and depression caused by various diseases, environmental changes and major changes. (4) Alcoholics and (or) psychoactive drugs, drug abuse or dependence.

### Research tools

#### Perimenopausal diagnosis

Referring to the relevant criteria for perimenopausal syndrome in the 9th edition of Obstetrics and Gynecology (18), the content of the Perimenopausal Symptom Questionnaire was designed independently to include: age, menarche, menstrual cycle and perimenopausal symptoms (memory loss, insomnia, anxiety, depression, menstrual disorders, fatigue, dizziness, palpitations, headache, tinnitus, vaginal dryness, recurrent vaginitis and urinary tract infection; urinary incontinence (straining, leakage after exercise or cough), nocturia, bone and joint pain, palpitations, chest tightness, precordial discomfort and other menopausal pseudoangina symptoms, menopausal hypertension caused by emotional instability, poor sleep, dysphoria, and metabolic abnormality-related symptoms (hyperlipidemia, hyperglycemia, weight gain, abdominal obesity, etc.).

#### Self-rating anxiety scale

The (19) was developed by Zung to assess the severity of an individual’s anxiety state over the past week. SAS is a simple clinical tool for analyzing subjective anxiety sensation in patients and is suitable for adults with anxiety symptoms, with the advantages of high effectiveness, simple method, and easy analysis. The scale consists of 20 items, with a 4-level score, 1 indicating “no or little time” and 4 indicating “most or all time.” The scores of each item are additive, and SAS ≥50 is considered anxiety, and the higher the score, the more severe the degree of anxiety. Cronbach’s alpha coefficient for this inventory was 0.820 (20).

#### Self-rating depression scale

The subjective experience of the (19) was developed by Zung to assess individuals’ depressive mood over the past week. SDS is a commonly used self-assessment tool for depression at home and abroad, which is characterized by easy use, intuitive and relatively accurate reflection of the degree of depression subjectively felt by self-assessors, and is a clinical tool suitable for adults to measure subjective depression feelings. The scale consists of 20 items scored at 4 levels, with 1 indicating “no or little time” and 4 indicating “most or all of the time.” The scores of each item were additive, standard score = coarse score × 1.25, less than 53 points for no depression, 53–62 points for mild depression, 63–72 points for moderate depression, and more than 72 points for severe depression. The higher the score, the more severe the depression. Cronbach alpha coefficient for this inventory was 0.84 (20).

#### Pittsburgh Sleep Quality Index scale

The PSQI (21) is a scale developed by the University of Pittsburgh to evaluate sleep quality in the past 1 month. This scale is used to assess individual sleep quality and consists of 7 sleep scoring factors, each scored on a scale of 0–3, and the cumulative PSQI total score range of each factor (0–21), with higher scores indicating worse sleep quality. The total PSQI score was divided into four levels: 0–5 points for good sleep quality, 6–10 points for better sleep quality, 11–15 points for fair sleep quality, and 16–21 points for very poor sleep quality.

### Study methods

#### Ethics approval

The studies involving human participants were reviewed and approved by Lyuliang University. Informed consent was obtained from all subjects.

#### Intervention process

From May 2022 to July 2022, aerobics intervention training was carried out in the community. A total of 289 subjects were divided into group training groups according to recruitment time, and considering the intervention training that can better guide each subject, and every 24–32 subjects were an intervention group. The Chengdong Community of Binhe Street carries out training every Monday and Wednesday afternoons, the Ecological Park Community of Fengshan Street carries out training every Tuesday and Thursday afternoons, and the Bayi Community of Lianhuachi Street carries out training every Friday and Sunday afternoons. During the experiment, the exercise items of each group were secondary mass aerobics (Four combinations in total, Combination 1:4*8*2. (1) Twice easy walk; (2) Take three steps forward/backward; (3) Twice V step; (4) 1–4 side crossing step 5–8 Twice parallel step; (5)–(8) The actions are the same as those in (1)–(4), but in the opposite direction. Combination 2: 4*8*2. (1). 1–2 The first half of the V step, 3–6 Hip swings left and right 4 times, 7–8 The second half of the V step; (2) Twice step suction leg; (3) 1–4 side crossing steps, 5–8 Left and right side point; (4) 1–4 Left suction leg twice, 5–8 Change in right leg; (5)–(8) The actions are the same as those in (1)–(4), but in the opposite direction. Combination 3:4*8*2. (1)/(2) Four parallel steps, “L” shape; (3) two-time meander; (4) Suction leg for 4 consecutive times in the upper step; (5)–(8) The movements are the same as those in (1)–(4), but in the opposite direction; Combination 4:4*8*2. (1) 1–4 Walk forward for 4 steps; 5–8 2 times of kicking; (2) Move backward with side parallel steps; (3) Hop Up and swing your legs twice, then mean; (4) Four step back bending, single and double; (5)–(8) The movements are the same as those in (1)–(4), but in the opposite direction.), and the duration of each exercise was 60 min (5 min warm-up, 10 min collation activities, and 45 min aerobics training), and the exercise intensity was controlled at 110–140 beats per minute with heart rate indicators, and 6 subjects were randomly selected each time using a Polar heart rate telemeter and heart rate was measured every 5 min. During the experiment, all subjects who participated in the experiment were asked to try not to participate in other physical exercises. In addition, subjects were instructed to perform self-practice at other times after class, and to record their number and time of practice each day.

#### Outcome measures

SAS, SDS and PSQI questionnaires were distributed on the spot, unified instructions were completed, answers were given before (baseline) and after the intervention, and subjects independently completed the questionnaire within 30 min. Before the intervention, 289 questionnaires were distributed and 289 valid questionnaires were returned, with a valid recovery rate of 100%. After the intervention, 289 questionnaires were distributed and 289 valid questionnaires were returned, with a valid recovery rate of 100%.

#### Statistical processing

The experimental data were statistically analyzed using the SPSS 20.0 statistical software package. Measurement data were expressed as mean ± standard deviation (Mean ± SD), Normally distributed and equal variance, *t*-test was used, and χ^2^ test was used to compare rates. *P* < 0.05 was considered statistically significant.

## Results

### Training completion

All study subjects completed 16 times aerobics sessions over 8 weeks. On this basis, 113 individuals practiced self-practice ≥3 times/week, 97 individuals practiced 1–2 times/week, and 79 individuals did not practice self-practice. The number of patients with anxiety, depression, and insomnia disorders included in different self-practice frequencies is shown in Table 1. Comprehensive statistics: self-practice ≥3 times/week, 57 anxiety (39.86% of the total anxiety), 59 depression (37.58% of the total depression), 66 insomnia (39.05% of the total insomnia); self-practice 1–2 times/week, 49 anxiety (34.27% of the total anxiety), 52 depression (33.12% of the total depression), 57 insomnia (33.73% of the total insomnia); no self-practice, 37 anxiety (25.87% of the total anxiety), 46 depression (29.3% of the total depression), 46 insomnia (27.22% of the total insomnia). All subjects filled out a complete checklist before and after training.

**TABLE 1:** Different self-practice in people with anxiety, depression and sleep disorders.

Group	A	D	SD	A and D	A and SD	D and SD	A, D and SD	Total
≥3 times/week	15	16	22	16	17	18	9	113
1–2 times/week	14	14	17	12	14	17	9	97
0	10	13	12	10	11	17	6	79
Total	39	43	51	38	42	52	24	289

A, anxiety; D, depression; SD, sleep disorders.

### Comparison of anxiety, depression and sleep status of subjects before and after intervention

All subjects completed a total of 16 group aerobics sessions over 8 weeks. After aerobics training intervention, anxiety, depression and sleep disorders of subjects were improved to varying degrees, SAS score, SDS score, PSQI score were significantly lower than before intervention, the score difference was statistically significant (*P* < 0.01) Table 2. Anxiety scores decreased in 119 of 143 (83.22%) anxiety subjects (*P* < 0.01); 132 of 157 (84.08%) depression subjects (*P* < 0.01); and 152 of 169 sleep disorder subjects (89.94%) (*P* < 0.01).

**TABLE 2:** Comparison of anxiety, depression and sleep disorders before and after intervention.

Scale	Number	Before intervention	After intervention	*t*	*p*
SAS scale	143	70.26 ± 6.9	45.72 ± 6.05	32.7	<0.01
SDS scale	157	71.28 ± 7.32	49.71 ± 7.67	27.28	<0.01
PSQI scale	169	19.99 ± 3.69	10.23 ± 4.94	21.03	<0.01

SAS, self-rating anxiety scale; SDS, self-rating depression scale; PSQI, Pittsburgh Sleep Quality Index.

### Symptom relief of subjects with different clinical manifestations

Among all 289 subjects who participated in the study, 261 had relief of symptoms, and the response rate reached 90.31%. Among them, those with only one symptom before the intervention were relieved after an 8-week aerobics training training intervention; those with both symptoms had a remission rate greater than 80%; and even those with both symptoms had a remission rate of more than 65% (Table 3).

**TABLE 3:** Symptom relief of subjects with different clinical manifestations.

Group	A	D	SD	A and D	A and SD	D and SD	A, D and SD	Total
Total (*n*)	39	43	51	38	42	52	24	289
Relief (*n*)	39	43	51	31	35	46	16	261
Rate (%)	100	100	100	81.58	83.33	88.46	66.67	90.31

A, anxiety; D, depression; SD, sleep disorders. Symptom relief criteria: two symptoms coexist, one of which is relieved; three symptoms coexist, two of which are relieved.

### Comparison of anxiety, depression and sleep of subjects under different exercise frequencies

On the basis of group practice, SAS score (*P* < 0.01), SDS score (*P* < 0.01) and PSQI score (*P* < 0.05) were significantly different after aerobics training intervention in patients who practiced autonomously ≥3 times a week, 1–2 times a week and those who did not practice autonomously. The higher the number of spontaneous exercises, the lower the anxiety, depression, insomnia scores (Table 4).

**TABLE 4:** Comparison of anxiety, depression and sleep of subjects under different exercise frequencies.

Scale	Group	Number	Before intervention	After intervention	*t*	*P*
SAS	≥3 times/week	57	71.05 ± 8.23	46.19 ± 7.48	26.35	<0.01
	1–2 times/week	49	71.49 ± 5.83	47.59 ± 6.61	32.88	<0.01
	0	37	71.26 ± 8.94	49.72 ± 4.06	26.97	<0.01
SDS	≥3 times/week	59	72.62 ± 8.50	49.71 ± 8.37	18.11	<0.01
	1–2 times/week	52	69.90 ± 3.91	51.71 ± 7.07	20.76	<0.01
	0	46	70.77 ± 3.58	53.33 ± 4.46	29.46	<0.01
PSQI	≥3 times/week	66	19.86 ± 5.81	10.19 ± 3.4	11.13	<0.01
	1–2 times/week	57	18.49 ± 2.45	11.53 ± 3.44	16.33	<0.01
	0	46	18.68 ± 2.40	13.04 ± 4.57	13.92	<0.01

Among the anxious subjects, 56 (98.25%) were relieved of symptoms and accounted for 47.06% of the total remissions in 57 subjects who practiced autonomously ≥3 times/week; 38 (77.55%) were relieved of symptoms and accounted for 31.93% of the total remissions in 49 subjects who practiced autonomously 1–2 times/week; and 25 (67.57%) were relieved of symptoms and accounted for 21.01% of the total remissions in 37 subjects who did not practice autonomously.

Of the depressed subjects, 58 (98.31%) had symptom relief and accounted for 46.4% of the total remissions in 59 subjects who practiced autonomously ≥3 times/week; 41 (78.85%) had symptom relief and accounted for 32.8% of the total remissions in 52 subjects who practiced autonomously 1–2 times/week; and 26 (56.52%) had symptom relief and accounted for 20.8% of the total remissions in 46 subjects who did not practice autonomously.

Among the subjects with sleep disorders, 66 subjects (100%) achieved symptom relief with spontaneous practice ≥3 times/week, accounting for 44.9% of the total remission; 51 subjects (89.47%) achieved symptom relief with 57 subjects (89.47%) with spontaneous practice 1–2 times/week, accounting for 34.69% of the total remission; and 30 subjects (65.22%) achieved symptom relief with 46 subjects (20.41%) without spontaneous practice.

The remission rates of anxiety, depression and sleep disorder symptoms were significantly different in subjects with different frequency of spontaneous practice, and the remission rates of the three symptoms were significantly higher in those with spontaneous practice ≥3 times/week than in those with spontaneous practice 1–2 times/week and those without spontaneous practice (*P* < 0.01); the remission rates of the three symptoms were significantly higher in those with spontaneous practice 1–2 times/week than in those without spontaneous practice (*P* < 0.01; Table 5).

**TABLE 5:** Comparison of remission rates of anxiety, depression and sleep disorders among subjects at different exercise frequencies.

Scale	Total relief (*n*)	≥3 times/week		1–2 times/week		0	
				
		Total (*n*)	Relief n (%)	Total (*n*)	Relief *n* (%)	Total (*n*)	Relief *n* (%)
SAS	119	57	56 (98.25)	49	38 (77.55)	37	25 (67.57)
SDS	132	59	58 (98.31)	52	45 (86.54)	46	28 (60.87)
PSQI	152	66	66 (100)	57	53 (92.98)	46	33 (71.74)

Yan Zhao: YZ and Hualiang Niu: HN.

## Discussion

This study showed that all perimenopausal women who volunteered to participate in the study were able to adhere to 16 aerobics training sessions over 8 weeks with good compliance, which may be related to the frequent occurrence of anxiety, depression and sleep disorders in the perimenopausal period without good intervention, or to the health care, medical treatment, fitness, bodybuilding, and interest and non-impairment unique to aerobics training. After the intervention, the symptoms of anxiety, depression and sleep disorder were improved to some extent, 83.22% of anxiety symptoms, 84.08% of depression symptoms and 89.94% of sleep disorders were relieved, indicating that aerobics training has a significant effect on improving the symptoms of psychological disorders in perimenopausal women. Aerobics training can be used as a safe and easy to operate training method to improve anxiety, depression and sleep problems in perimenopausal women.

### Anxiety, depression and sleep quality in perimenopausal women

Perimenopause represents a delicate transitional period of female life during which physical, emotional, psychological, and social changes mark the progression of women from fertile life to climaterium or reproductive loss, withwide sexual fluctuations until the onset of hypergonadotropic hypogonorrhea (22). It is generally 45–55 years of age and is an important stage in the transition of women from middle to old age. Although neuroendocrine changes during the menopausal transition are varied and complex, the central biological event during this period is gradual ovarian failure, both in the number of follicles and in the quality of oocytes. Because ovarian function gradually deteriorates, causing dysfunction of the hypothalamic-pituitary-ovarian endocrine axis and decreased estrogen levels, about two-thirds of women experience varying degrees of perimenopausal symptoms (23–25). For example, autonomic dysfunction symptoms (palpitations, vertigo, headache, insomnia, tinnitus; skin paresthesia, etc.) (26–28) and psycho-psychological symptoms (irritability, anxiety, depressed mood; memory loss, cognitive decline; depression, inability to self-control mood, etc.) (29–31) as well as cardiovascular system symptoms (menopausal pseudo angina symptoms such as palpitations, chest tightness, precordial discomfort; menopausal hypertension due to emotional instability, poor sleep, dysphoria, etc.) (32–35) directly affect women’s physical and mental health and well-being. Investigations have shown that adult females have 41% higher insomnia rates than males (36). Before puberty, there is no male-to-female difference in the prevalence of insomnia, but with menstruation, the rate of insomnia increases dramatically in women, 2.5 times that in men (37). Before and after natural menopause, the incidence of insomnia disorder in women increased significantly compared with that before menopause. According to the National Women’s Health Study (SWAN), the prevalence of sleep disorders increases with age. The prevalence of sleep disorders varies from 16 to 42% in premenopausal women, 39 to 47% in perimenopausal women, and 35 to 60% in postmenopausal women (38). Lee et al. (39) stated that perimenopausal sleep problems are distinct from other causes of sleep disorders and are mainly characterized by: difficulty falling asleep, frequent awakenings at night, early morning awakenings, and inability to sleep after awakening. In addition to sleep disorders, women entering the perimenopausal period experience a range of neuropsychiatric changes due to alterations and dysregulation of the body’s associated endocrine function, causing psychological dysfunction, mainly manifested as depression and anxiety, and often both symptoms coexist (40). A survey of epidemiology in China showed that perimenopausal women experienced anxiety in 81.58%, mild to moderate anxiety in 68.42%, depression in 85.09%, and mild to moderate depression in 73.68% (41). Mehrnoush et al. (42) showed that menopause is not a high-risk period for mental illness but causes psychological problems, the most common of which are anxiety and depression, which impair their ability to cope with events in daily life and work, and reduce the quality of life of women. Herson et al. (43) have shown that women not only have an increased risk of depression and anxiety during the perimenopausal and menopausal transition (MT) periods, but also experience higher severity of depressive symptoms during the perimenopausal period compared with before and after menopause. The latest Australian mental health data show that mental illness is more prevalent in women than in men, and that women who develop major depression are twice as likely as men (44). However, recurrent endocrinological changes before and after menopause may be responsible for the higher risk of depression from adolescence to old age in women than in men (45). The term anxiety is commonly used to describe a wide variety of symptoms and may include features of various anxiety disorders, such as panic disorder (e.g., sudden feelings of unexplained fear) or generalized anxiety (e.g., excessive and uncontrollable worry, irritability), or physical symptoms, such as shortness of breath and rapid heartbeat. Relatively few studies have been conducted on the relationship between anxiety and menopause (46). However, studies have shown that the menopausal transition is a time of increased risk for the onset or worsening of anxiety symptoms (47), and studies have also demonstrated a significant increase in anxiety symptoms in the perimenopausal period (48).

### Effect of exercise on anxiety, depression and sleep quality in perimenopausal women

Exercise, as a low-cost, uncomplicated, and non-invasive non-pharmacologic therapy, not only helps prevent and improve many health problems, such as lowering blood pressure, enhancing cardiovascular fitness, losing weight, and preventing chronic diseases, such as cancer, diabetes, hypertension, obesity, osteoporosis, musculoskeletal pain, and neurodegenerative diseases (Parkinson’s disease, Alzheimer’s disease, and amyotrophic lateral sclerosis, etc.) (49, 50), but also helps improve mental disorders (such as anxiety and depression) and reduce the risk of morbidity (51). In otherwords, exercise promoteswell-being and enhancing mood. Studies have shown that working out and other forms of physical activity have irreplaceable promoting effects on human physical and mental health (52).

Studies have shown that the use of exercise to treat major depression has been recommended in some clinical guidelines (53), suggesting that exercise improves overall health, including depressive symptoms inwomen in their middle-aged and olderyears, according to a recent meta-analysis (54). Similar findings were obtained by Roh (55), where a 16-week Pilates exercise program resulted in significant relief of depressive symptoms in older women. For anxiety, adults over the age of 50 with high levels of physical activity report lower levels of anxiety symptoms and status (56). In a recently published meta-analysis, itwas found that programmed exercise, at a low-to-moderate intensity for at least sixweeks, seems to improve symptoms of mild to moderate anxiety in middle-aged and elderlywomen (57). In addition, Li et al. (58) showed that a 3-month fitness running exercise intervention (3 times/week, 50–70% maximum heart rate) resulted in a significant improvement in depression in depressed women, as shown by a significant decrease in depression handsome selection scale scores, self-rating depression scale scores, and depression 90-item symptom checklist scores. A study by Tiller (59) of Australian women showed that women who were less than the recommended 2.5 h of moderate exercise per week continued to experience higher rates of anxiety and depression. Aibar-Almazán et al. (60) analyzed the effects of Pilates based exercise program on sleep quality, anxiety, depression, and fatigue in postmenopausal women aged 60 years and older living in Spanish communities, and showed that 12-week Pilates exercise intervention had beneficial effects on their sleep quality, anxiety, depression, and fatigue. Specifically, all Pittsburgh Sleep Quality Index (PSQI) domains aswell as in the PSQI total score were significantly improved, and Hospital Anxiety and Depression Scale (HADS) score and fatigue severity scale were significantly reduced. Poor sleep quality is strongly associated with a low level of physical activity among postmenopausalwomen (61). Cai et al. (62) study showed,traditional exercise training programs, such as aerobics, it has been shown to positively affect on sleep quality in postmenopausal women, presented as10-week moderate- to high-intensity step aerobics training program (40–45 min/times, 3 times/week, 10 weeks, intensity of 75–85% of the heart rate reserve)can enhance the quality of sleep and increase the melatonin levels in sleep-impaired postmenopausal women, and a systematic review and meta-analysis of randomized controlled trial concluded that programmed exercise enhance sleep quality among middle-aged women (63). Tadayon et al. (64) also found improvements in both the Pittsburgh Sleep Quality Index total score and domains among postmenopausal women after twelve weeks of pedometer-based walking. Newton et al. (65) randomized controlled studies performed showed that, postmenopausal women with insomnia were randomly assigned to either yoga classes, exercise at home, or their usual activity. They found that, compared to usual activity, women assigned to yoga saw improvements in their sleep quality and sleep disorders, both domains of Pittsburgh Sleep Quality Index. Buchanan et al. (66) studie showed that, 12 weeks of yoga or aerobic exercise can makelate transition and postmenopausal women aged 40–62 years with hot flashes Significant improvement in sleep stability, mean total sleep time markedly increased,mean wake after sleep onsetmarkedly decreased, mean coefficient of variationfor number of long awakenings >5 min markedly decreased. Curi et al. (67) reported improvements in Pittsburgh Sleep Quality Index total score, use of sleeping medication, and sleep latency after 16 weeks of intervention with Pilates exercises in women aged 60 years and over. Gulia and Sreedharan (50) confirmed the research on post menopausal subject, after administering the 24 weeks of yoga-nidra practice and exercise module protocol, there was remarkable elevation in mood both on waking up and entire day from 5th week onward. Mood shifted toward a happier state. Latency to sleep decreased after 4 weeks, while total sleep time improved only after 16 weeks of dual management strategy.

### Effect of aerobics training on anxiety, depression and sleep quality in perimenopausal women

As an aerobic exercise, aerobics with its enthusiasm, rhythm, movement stretch, beautiful and powerful, giving people booming, spiritual quality, free driving force, so that exercisers and ornaments can feel the beautiful realm of the combination of aerobics and beauty. It is very rich in physical exercise and mental health care value, with popularity, the characteristics of the whole people, suitable for the diverse needs of Chinese public fitness, but also an important part of the national fitness exercise. Completing a set of aerobics is equivalent to doing a thorough whole body movement. Aerobics can improve and improve the motor function of muscle fibrous tissue, joint tissue and bone. Mass aerobics can also be flexibly arranged for different objects, different purposes of exercisers (such as weight loss, strong waist, body, etc.). For example: middle-aged and elderly aerobics, adolescent aerobics, young children aerobics, pregnant women aerobics, warm-up aerobics, body aerobics, rhythm aerobics, weight loss aerobics, free-hand aerobics, device aerobics, body parts aerobics and so on. Moreover, mass aerobics strictly follow the physiological law of sports load from small to large, movement from simple to complex; intensity from weak to strong, gradually increase load, and gradually decrease when reaching and maintaining a certain negative. In short, mass aerobics can enhance physical fitness, improve body shape, change mental outlook and temperament, purify the mind, give people beauty edification. According to the researchers, women participating in mass aerobics can not only maintain a positive and optimistic emotional state and improve mood, but also have a certain intervention effect on depression and anxiety. Beautiful and cheerful music rhythm, lively and happy body movements can maximize the enthusiasm and vitality of people, so that the tired mind can be effectively relaxed, physical and mental regulation. For women who concentrate on exercise, under the guidance of professional coaches, they can not only get systematic fitness exercise, enhance self-confidence, but also enhance sense of honor and collectivism, which helps to establish a good interpersonal relationship. In this study, after 8 weeks of aerobics training, the anxiety, depression and sleep status of perimenopausal women were significantly improved, indicating that aerobics training can be used as a relatively safe and easy to operate training method to improve the anxiety, depression and sleep problems of perimenopausal women. The results of this study also showed that those who had only one symptom of anxiety, depression and insomnia before the intervention, all symptoms were significantly relieved after the intervention. The remission rate was more than 80% when the two symptoms existed before intervention. Even those with the three symptoms at the same time had a remission rate of over 65 percent. It indicates that even if the symptoms are more complicated and the disease is more serious, the 8-week aerobics training can also significantly alleviate. According to the frequency of self-practice after class, it was found that the more the number of self-practice of aerobics training, the lower the SAS score, SDS score and PSQI score, indicating that the number of training, frequency and amount of training can affect the intervention effect. Further statistics showed that anxiety was relieved by 98.25%, depression was relieved by 98.31%, and sleep disorder was relieved by 100% for those who exercised by themselves more than 3 times a week. The remission rates of anxiety, depression and sleep disorders were 77.55, 86.54, and 92.98% for those who practiced by themselves 1–2 times a week. However, the relief rates of the three symptoms were 67.57, 60.87, and 71.74% for those who only relied on collective training and did not exercise by themselves, showing significant differences between the groups, indicating that even short-term concentrated calisaerobics training can also play a positive role in alleviating anxiety and depression symptoms and improving sleep.

## Summary

Aerobics training can effectively improve the anxiety and depression of perimenopausal women, improve the quality of sleep, and can be used as a predictive method to improve the mental health level of perimenopausal women. This method requires simple conditions, can use group counseling, can also be independent practice, is an effective psychological intervention means, suitable for clinical promotion.

Mental health intervention for perimenopausal women is a long process. Although this study found that the SAS score, SDS score and PSQI score of perimenopausal women were improved to a certain extent after 8 weeks of intervention, further continuous intervention and follow-up verification are needed to systematically evaluate the long-term efficacy of aerobics training intervention. In addition, the sample size included in this study was not large enough, and subsequent work needs to expand the sample size to verify these conclusions. And when it comes to why exercise can improve mood and mental health, These include the endorphin hypothesis, the thermogenic hypothesis, mitochondrial dysfunction, mammalian target of rapamycin (mTOR), neurotransmitter dysfunction and the hypothalamic pituitary-adrenal (HPA) axis, All of which have been proposed to play a mechanistic role in altered mental states. Further studies are needed in the future to investigate the physiological and biochemical molecular mechanisms by which exercise improves depression, anxiety, and sleep disorders in perimenopausal women.

## Data availability statement

The original contributions presented in this study are included in the article/supplementary material, further inquiries can be directed to the corresponding author.

## Ethics statement

The studies involving human participants were reviewed and approved by Lyuliang University. The patients/participants provided their written informed consent to participate in this study.

## Author contributions

YZ: writing—original draft preparation. HN: data collection. SJL: writing—review and editing. All authors read and agreed to the published version of the manuscript.
